# Relationship between female employees and firm’s innovation: Evidence from Japanese companies

**DOI:** 10.1371/journal.pone.0323751

**Published:** 2025-06-03

**Authors:** Taro Ichikawa, Mika Goto

**Affiliations:** School of Environment and Society, Institute of Science Tokyo, Minato-ku, Tokyo, Japan.; Jinan University, CHINA

## Abstract

This study investigates how female employees contribute to firm innovation by applying panel data Poisson regression models to data pertaining to 144 major Japanese manufacturing firms from 2004 to 2022. The results reveal the following: First, there is a positive relationship between longer female employment and innovation. Longer female employment may be attributable to female employees feeling more secure at their workplaces. Second, regarding women’s unique perspectives, this study suggests that female participation in innovation may increase the number of technological fields. Third, this study presents the mediating effects of technological fields on the relationship between high-impact innovation and the demographic of female employees. These findings suggest corporate management creates a system that allows female employees to play an active role in the workplace with long-term employment. The empirical results provide new insights into previous literature on corporate innovation, specifically technological innovation.

## 1 Introduction

Starting in the 1950s, Japan’s high economic growth was driven by the manufacturing industry, and its male-centered social structure supported a growth model of mass production and price competition [1]. Based on the expectation of the husband’s long-term stable employment in exchange for long working hours, men received the benefits for sacrificing their private lives and devoting themselves selflessly to the company, and women received some allowances for giving up their own careers to support those of their husbands [2]. However, around the year 2000, this period of high growth had passed and Japan began to experience global competition, deflation, a declining birthrate, and an aging society. As the cost-benefit balance of the male-breadwinner family model worsens, Japanese companies are reducing their core employees, i.e., men, and expanding their periphery labor force, i.e., women [2–4].

To strengthen gender equality, the revised Equal Employment Opportunity Law came into effect in 1999 [5]. In the 2000s, as firms enhanced personnel systems supporting work-life balance, and working environments became more conducive to the use of such systems, employment continuity for women improved to a certain extent. However, there is a very limited number of women in managerial positions or on the boards of directors [6]. Men still comprise most of the top management teams (TMT) in Japanese companies, and the rate of Japanese female executives is among the lowest (9.1% in 2022) in developed countries [7]. It is noted that the percentage of female directors and executive officers (including directors, executive officers, and corporate auditors) is as of July 2022, according to “Yakuin-Shikihou, 2023.” Japanese companies have a closed culture designed to maintain organizational harmony within departments or specialized areas. This closed culture fosters a corporate culture that excludes outsiders [8].

Although the amount of R&D investment made by Japanese companies, which supports their growth, is not far behind that of Western companies, the profitability of Japanese companies is far below that of their Western counterparts [9]. A model of Schumpeterian growth analysis implies that the slow productivity growth of the Japanese economy since the 1990s may have been compounded by lackluster economic reform and insufficient innovation [10]. Major innovation can be promoted by increasing employee diversity and their interactions with each other [11]. Thus, the Japanese government has announced that female employees are urgently needed to accelerate innovation in Japanese companies [12]. However, Japanese corporate executives are still skeptical about whether women’s contributions are truly helpful in creating innovation [13].

Most previous studies on innovation creation have focused primarily on the diversity of TMT levels, such as CEO or CTO, based on the idea that diversity in top leadership is important for innovation (i.e., upper management theory; [14–18]). Although research on the impact of employee diversity on innovation is limited, especially for Japan [19,20], employees in positions below the leadership level should have an important impact on innovation [21].

Therefore, this study focuses on gender diversity among employees. The purpose of this study is to investigate through quantitative analysis how female employees could contribute to corporate innovation, specifically technological innovation in the manufacturing industry, which drives Japan’s high economic growth [22]. Given the stagnation in economic growth that occurred until the 1980s, the subsequent lost decades of the Japanese economy [23,24], the novelty of this study is its use of Japanese manufacturing industry data from the 2000s onward. Moreover, we analyze the potential effect of female employees, rather than TMT, on innovation. The study’s implications will call the attention of Japanese managements to the importance of encouraging the long-term employment of female employees and promoting diversity management. Consequently, we hope this will lead to the realization of sufficient technological innovation and high profitability of Japanese companies.

This study analyzed 144 manufacturing firms listed on the Nikkei Stock Average as of July 2023. Panel data of each firm for 2004–2022 were created. Therefore, this study used approximately 2100 firm-year observations.

The remainder of this paper is organized as follows. Section 2 summarizes the literature on female employees and innovation, discusses the theoretical background, and develops the hypotheses. Section 3 presents the database and descriptive statistics. Section 4 tests the hypotheses regarding female employees and patents as an indicator of innovation. Section 5 presents the discussion and conclusions, recommendations for management, and limitations of the study.

## 2 Related studies and hypotheses

### 2.1 Relationship between TMT diversity and innovation

Traditionally, upper-level theory [25,26] is based on the assumption that TMT is critical for innovation, and numerous studies have focused on the CEO, CTO, or other TMT members.

First, the CEO is considered more influential than other executives in the innovation process [27,28]. Therefore, the relationship between CEOs and innovation has been studied from multiple perspectives, including CEO age [29,30], educational background [31–35], personality [36–39], R&D experience [40,41], and biography [42,43].

Next, several studies have focused on the attributes of TMT members, such as the number of TMT members [44], race [16], gender [45,46], ratio of outside directors [45], R&D experience [19,47,48], age [27] and so on.

The role of the CTO is becoming increasingly important within the TMT [49–51]. CTOs are involved in CEOs’ innovation decisions [52,53]. Additionally, many studies have shown that CTOs positively impact firms’ innovation activities [54].

As more women have entered TMT positions in recent years, many studies have shown positive relationships between female leaders and innovation [14,46,55]. In particular, female CTOs are more likely to innovate than are male CTOs [15,18].

### 2.2 Relationship between employee diversity and innovation

Just as diversity among TMT promotes innovation, diversity among employees is also expected to promote innovation. Previous studies conducted outside Japan have found a positive relationship between employee diversity and innovation [56–60]. Some studies suggest that gender diversity at the employee level is more conducive to innovation than it is at the management level [21]. However, employee diversity has been considered a double-edged sword [61]. For instance, one study noted that too much diversity can lead to a lack of communication, which can create conflict and distrust within organizations [62]. Other studies have shown that the relationship between gender diversity and innovation is limited or not significant [63,64]. Further research is required to determine how female employees in non-managerial positions affect innovation creation. Moreover, most studies on the relationship between employee diversity and innovation have been conducted in Europe, the U.S., China, and India [11], while few have focused on Japan [19,20].

Owing to the strong family culture in Japan, the Japanese firm tends to be regarded as a family, and management and employees operate as a community of shared destiny [65–67]. According to an independent survey of Japanese firms, a significantly higher ratio of large firms has chosen the “bottom-up approach” compared to small- and medium-sized firms [65]. In addition, studies have also shown that Japanese firms have been increasing productivity by involving employees in the decision-making process and increasing trust between management and employees [68]. Therefore, employees should have some influence on innovation. However, it remains unclear whether the analysis results for gender diversity outside Japan can be similarly applied to Japanese firms, or if they cannot be applied due to Japan’s strong patriarchal society.

Japan has the highest level of masculinity globally, which is measured by Hofstede’s cultural dimension [69]. As a result, a large wage gap exists between males and females, and females are less likely to be employed full-time and are promoted more slowly [70,71]. In addition, males with high school diplomas reach management positions faster and earn higher wages than do females with college degrees because of males’ low participation in household chores and females’ inability to work long hours [72,73]. In fact, 70%–80% of female employees in Japanese firms leave their jobs for family-related events, such as pregnancy or childbirth [74]. Consequently, many females are unable to return to their original firms after giving birth, and are relegated to part-time employment [75]. Another report found that female scientists are more likely to be single and must wait longer to be promoted in the workplace [76]. Given this background, a very limited number of women is in managerial positions or on the boards of directors [6,77,78], which might imply that female employees are not as active in Japanese firms as are their male counterparts. This situation has been pointed out as a factor in the sluggish profitability of Japanese firms and their declining ability to generate innovation [9].

In response to this situation, since 2014, government policies have repeatedly emphasized the importance of promoting women’s activities [12,79,80]. As it is necessary to verify whether promoting women’s activities actually promotes innovation, we use the average years of employment as an indicator of whether women are actually active [70,81]. If the ratio of female employees is used as an indicator of female advancement, it is impossible to eliminate the influence of management that increases the ratio of female employees due to female employees’ low wages [120]. According to the 2010 Survey of Gender Equality in Employment Management, 31.2% of companies with at least 1,000 employees felt that the fact that “the length of continuous service of women is short on average” is a problem in promoting greater participation by women [74]. If women stay longer in their firms, the probability of family events such as marriage, childbirth, or house affairs will get higher, and then they could be disadvantaged in the firm or eventually be pressured to resign. However, if a firm has longer average employment of women, that would mean women are not severely disadvantaged to the extent they give up their career by the family events and they have chance to play an active role in the firm on an equal footing with men. We clarify this effect between the length of employment years of female employees and its impact on innovation by developing the following hypothesis.

**Hypothesis 1** A positive relationship exists between years of female employment and innovation.

We believe that Hypothesis 1 is novel because it uses years of female employment as an indicator of female employee activities, rather than the number of female employees or the ratio of female employees used in previous studies [21,56,58], and analyzes the relationship with innovation for the first time. For a more detailed comparison between the ratio of female employees and years of female employment in Japan, see Appendix A. Note that female employees are defined as full-time female employees, excluding the members of the board of directors. Boards of directors in Japanese companies are often nominated by internally promoted directors from among employees [121]. Therefore, management and execution are not as separate as in Western companies [122]. To move closer to the global standard, The Fifth Basic Plan for Gender Equality in 2018 set a target of 12% by 2022 for the percentage of women on the boards of directors of companies listed on the first section of the Tokyo Stock Exchange [79]. In fact, as of July 2022, the ratio was 9.1%, falling short of the target. The first reason why we do not focus on female executives in this study is that the number of female executives in Japanese firms to date has itself been small. In the companies covered in this study, the observed number of female directors in the engineering field is almost nonexistent at 0.6%, and the number of female CTOs is 0. Second, Japanese firms tend to nominate female executives and outside directors because of impression policies [13,123].

### 2.3 Relationship between female employees, the scope of technological research, and high-impact innovation

**Fig 1:**
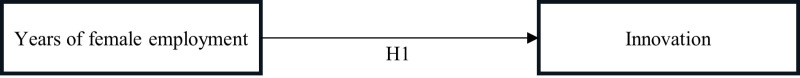
Conceptual model of Hypotheses 1.

Firms with more technological holdings have been found to remain innovative and are long-term performers [82,83]. Technological diversity and gender diversity are known to have a positive impact on innovation outcomes [84]. Women’s diverse perspectives, communication skills, and team orientation may impact the expansion of technological fields. One study has pointed out that female researchers and male researchers have different research interests [85]. In fact, there are known examples of successful product innovation and process innovation unique to the female perspective that could not be achieved by men only [9,86]. In addition, a study on the gender of inventors in patent applications in Spain has shown that patents that include women have a greater number of inventors than do patents that only include men [87]. This trend has been observed in all technological fields. Mauleón and Bordons point out that this is because men are oriented toward individual achievement, while women are oriented toward collective achievement. Based on the above findings, women’s involvement in innovation creation is expected to bring new female perspectives to the technological fields that have traditionally been dominated by men. Moreover, women’s team-oriented approach could increase the number of inventors, thus bringing a fresh perspective. Since the perspectives may correspond to a particular technological field, we formulate the following hypothesis, whose concept is shown in Fig 1.

**Hypothesis 2-1** A positive relationship exists between years of female employment and the breadth of technological field of innovation.

Hypothesis 2-1 is novel because it represents the first ever proposal of the existence of a relationship between the degree of female employees’ activities and the breadth of technological field of innovation.

**Fig 2:**
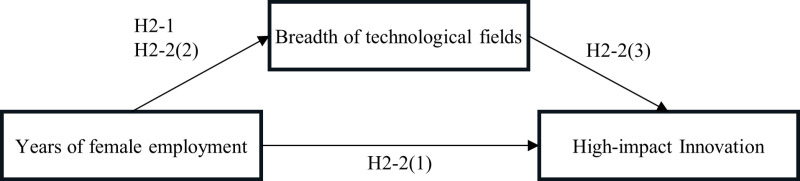
Conceptual model of Hypotheses 2-1 and 2-2.

The broad extent of the technology field is known to be more favorable for exploratory innovation [88] or radical innovation [89]. If the breadth of the technological field expands with the participation of female employees according to the above hypothesis, there must be a mechanism for the promotion of exploratory innovation or radical innovation as well. In fact, the presence of female CTOs in the US manufacturing industry has been shown to be positively correlated with exploratory innovation [18]. However, to the best of our knowledge, little is known about the correlation between exploratory/radical innovation as high-impact innovation and female employees. Therefore, we develop the following hypothesis for years of female employment, breadth of technological fields, and high-impact innovation as shown in Fig 2.

**Hypothesis 2-2** The breadth of technological fields mediates the relationship between years of female employment and high-impact innovation.

Hypothesis 2-2 is novel because it is the first ever proposal for why female employees’ active participation is related to high-impact innovation, and the possibility that this relationship is mediated by the breadth of technological field, providing the reason for female participation.

## 3 Data & Research Methods

### 3.1 Samples and Data

In this study, we focus on the manufacturing industry and select 144 manufacturing firms listed on the Nikkei Stock Average as of July 2023 (Table 1). We observe approximately 2700 firm-year data from 2004 to 2022. Based on the OECD’s 2020 R&D expenditure data, our selected industries cover about 88% of Japan’s total R&D expenditures. The R&D expenditure data of each industry in Japan as of 2020 are obtained from “OECD.Stat.” Organisation for Economic Co-operation and Development. Accessed January 2, 2024. https://stats.oecd.org/. Industries that do not fall into the manufacturing sector, such as finance, services, transportation, and energy, are excluded. This is because they have either low R&D expenditures or a low number of patent applications.

**Table 1:** Firms analyzed in this study by industry code.

Code	Industry	Number of companies	Obs.
4	Fisheries	1	19
5	Mining	1	16
6	Construction	9	171
9	Foodstuff	11	203
11	Fibre	2	38
14	Pulp & Paper	2	29
15	Printing	3	55
16	Chemistry	17	322
16a	Pharmaceutical	9	164
17	Petroleum	2	30
19	Rubber	2	38
21	Kiln industry	8	152
22	Iron and steel	4	76
23	Non-ferrous metals	8	151
27	Machine	16	304
28	Precision equipment	6	114
29	Electrical equipment	30	569
30	Shipbuilding	2	38
31	Automobile	10	190
32	Others	1	19
	Total	144	2698

For information on the employees of each company (age of employees, gender, years of employment, etc.), we use a Toyo Keizai Inc. CSR database. Toyo Keizai Inc. provides a CSR database, which is widely recognized and used in Japan. The database has been constructed by surveying each company since 2005, and is published yearly. “CSR Kigyou-Soran. (in Japanese)” Toyo Keizai Inc. Accessed July 2, 2024. https://str.toyokeizai.net/databook/dbs_csr_emp/. We obtain data from 2004–2022. For patent information as an indicator of innovation, we use the PatentSQUARE database. Panasonic provides the PatentSQUARE database covering all Japanese patents issued after 1983. This patent database is constantly updated. The data are obtained from April to June 2024. “PatentSQUARE.” WIPO. Accessed January 2, 2024. https://inspire.wipo.int/patentsquare. We obtain data from 1997–2022. Attribute information on TMT, such as gender, age, educational background, and duties, is obtained from the Annual Securities Report (the “Yakuin-Shikihou”), and Internet information for the period 2004–2022. Toyo Keizai Inc. provides a database of executives, which is widely recognized and used in Japan. The database has been constructed by surveying each company since 1983, and is published yearly. “Yakuin-Shikihou.” Toyo Keizai Inc. Accessed July 2, 2024. https://str.toyokeizai.net/magazine/yakuin/. Financial information for each company is obtained through annual reports covering the period 2004–2022. Finally, about 2100 firm-year observations are available due to an unbalanced panel dataset. CSR data are available only from 2004 and some data are missing. We perform no imputation for the missing data, therefore observations having missing value(s) are excluded from this analysis.

### 3.2 Methods

#### 3.2.1 Innovation Indicators.

In accordance with previous studies, four types of indicators are used [42,90]. The first indicator is the number of patent applications (*Patent Applications*), which is the total number of patent applications filed by a given firm in a given year. The number of patent applications provides an estimate of the amount of innovation such as successful R&D efforts [91,92]. It is noted that any R&D effort with no patent application (i.e., the research effort “failed”) is not detected in this study. Next, to assess innovation quality, we count the number of patents granted (*Patents Granted*) registered by the Japan Patent Office (JPO). Focusing on patents granted, it is possible to assess the novelty and inventiveness of the patents [93]. Next, we count the number of total citations (*Citations*) of the patent applied for. The number of citations can be used to evaluate the degree to which the patent has impacted the firm inside and outside [94–96]. Finally, we count the number of outside citations (*Outside Citations*), which is the number of citations excluding the number of self-citations. The number of outside citations can indicate whether the innovation is new to a firm and impacts other firms.

#### 3.2.2 Gender Gap Variable.

Average years of female employment are used for the gender gap variables (*Years of female employment*)*.* The data are reported yearly starting from 2004 by the publisher Tokyo Keizai Inc., based on a questionnaire or interview with each firm concerning human resource development, environment measures, corporate governance, and social relations. We also obtain average years of male employment in the same way, and use for control variable (*Years of male employment*). Data overview is shown in appendix A.

#### 3.2.3 Control Variables.

Various firm-level variables are used as control variables. To focus on R&D expenditures that directly affect innovation, we use the ratio of R&D expenditure to total assets (*R&D intensity*). Increased R&D spending implies greater innovation [97]. In addition, we use the natural logarithm of total employees (*Firm Size*) because the likelihood of introducing innovations increases with scale [98]. To control for the impact of a firm’s growth potential and financial constraints on innovation, we used the natural logarithm of years the firm has been listed on the Tokyo Stock Exchange (*Firm Age*), the ratio of net income to total assets (*ROA*), the ratio of tangible fixed assets to total assets (*Tangibility*) and the ratio of fixed debt to total assets (*Leverage*) [18].

To control for the impact of TMT, we set the natural logarithm of directors age as the directors’ attributes (*Directors age*). Directors’ age has a negative association with R&D spending [99]. Next, as a factor of CTO, we set a dummy variable of 1 if the company has a CTO on the board of directors and 0 if not (*CTO*). Since the impact of TMT diversity on innovation is known, we also set the *Outside Directors Ratio* (*Outside Directors Ratio*) and the *Female Directors Ratio* (*Female Directors Ratio*) as in previous studies [16,100]. The auditors among the board members are not included in the measurements.

#### 3.2.4 Other Variables.

Several indicators are used to assess whether female employees broaden the technological fields when innovating. First, the inventors involved in a single patent application are counted, and then the total inventors in a given firm in a given year (*Inventors*) are calculated. The more inventors there are, the more likely inventors’ technical backgrounds will be collected. Second, we measure the number of technological field codes for each patent application. The number of FI (*FIs*) is the JPO’s technological field code finetuned from the original IPC code. Each FI represents one technology area, and a higher number of FIs indicates that more technology fields are involved in the innovation process [83,101,102]. Third, the number of theme codes (*Themes*) that are expressed into five-digit alphanumerical codes are used. This is used in JPO for uniquely dividing patent documents into technical fields [101,103]. Fourth, we measure the number of claims (*Claims*) [100]. Additional claims indicate that more technological aspects are being considered in innovation [104].

We introduce a metric to assess whether female employees generate high-impact/quality innovation. Referring to previous study [18], if the number of outside citations of a patent application exceeds 90% of all citations, the patent application is classified as a high-impact patent application (*High-impact Patents*)

Table 2 summarizes the variables.

**Table 2:** Variables used in this study.

Variable	Description	Source
***Innovation variables***		
Patent applications	Number of all patents filed in the relevant year	PatentSQUARE
Patents Granted	Number of patents that were finally granted out of the patents applied for in the relevant year.	PatentSQUARE
Citations	Total number of citations received by the firm and other firms for patents filed in the relevant year.	PatentSQUARE
Outside Citations	Total number of citations received from other firms for patents filed in the relevant year	PatentSQUARE
***Gender Gap variable***		
Years of female employment	Average years of employment of female employees	Toyo Keizai CSR Firm Directory
D_HighFemaleYE	Dummy variable if the gap between female and male years employment is smaller than the total average gap (2.63 years), it is set to 1, otherwise 0	Toyo Keizai CSR Firm Directory
***Control variables***		
Firm size	Natural logarithm of total employees	Annual Securities Report
Firm age	Natural logarithm of years since the first appearance at Tokyo Stock Exchange	Annual Securities Report
ROA	Ratio of net income to total assets	Annual Securities Report
R&D intensity	Ratio of R&D expenditure to total assets	Annual Securities Report
Tangibility	Ratio of tangible fixed assets to total assets	Annual Securities Report
Leverage	Ratio of fixed debt to total assets	Annual Securities Report
Female ratio	Ratio of female employees	Toyo Keizai CSR Firm Directory
Years of male employment	Average years of employment of male employees	Toyo Keizai CSR Firm Directory
Directors age	Natural logarithm of directors’ average age including CEO	Annual Securities Report
CTO	1 if there is a CTO on the board of directors, 0 otherwise.	Annual Securities Report, Quarterly Report of the Board of Directors
Outside Directors ratio	Ratio of outside directors to all directors	Annual Securities Report,Quarterly Report of the Board of Directors
Female Directors ratio	Ratio of female directors to all directors	Annual Securities Report,Quarterly Report of the Board of Directors
Year Dummy	Dummy variables from year 2004–2022	All
Industry Dummy	Dummy variables from industry 4–32	All
***Other variables***		
High-impact Patents	Number of patents for which prior art citations from sources other than the firm exceed 90% of the total number of patents filed in the relevant year.	PatentSQUARE
Inventors	Number of inventors for patents filed in the relevant year	PatentSQUARE
FIs	Total number of JPO’s technical fields (File Index) for patents filed in the relevant year	PatentSQUARE
Themes	Total number of JPO’s theme codes for patents filed in the relevant year. Theme codes are five-digit alphanumerical codes for dividing patent documents into technical fields	PatentSQUARE
Claim	Total number of claims for patents filed in the relevant year	PatentSQUARE

### 3.3 Estimation model

The estimation model used in this empirical analysis is a panel data Poisson regression model with fixed effects because we treat count data as a dependent variable. The Poisson distribution is appropriate for a dependent variable *y* that takes only nonnegative integer values: {0, 1, 2, …}. It can be widely used to model the number of occurrences of an event, such as the number of patent applications by a firm by a year. The probability density function of the Poisson distribution is expressed as follows [105].


$$f\left(y_{it}|\lambda_{it}\right)=\frac{e^{-\lambda_{it}}{\lambda_{it}}^{y_{it}}}{y_{it}!},\;$$ (1)

where $y_{it}$ is the dependent variable for the *i*-th observation in the *t-*th period, an integer greater than or equal to 0. $\lambda_{it}$ is expressed by the following relationship with an expected value E and a variance Var,


$$E\left[y_{it}\right]=Var\left[y_{it}\right]=\lambda_{it}.\;$$ (2)

In the Poisson regression model, parametrization takes the form of an exponential function, as shown in the following Equation (3):


$$\lambda_{it}=\alpha_{i}\exp\left(x_{it}^{\prime }\beta \right),\;i=1,...,N,\;t=1,...,T,\;$$ (3)

where $x_{it}^{\prime }$ is the independent variable vector for the *i*-th observation in the *t-*th period; $\alpha_{i}$ is the time-invariant fixed effects for i=1,...,N, and *β* is the regression parameter vector. Equations (4) and (5) indicate the logarithmic link functions.


$$\ln\left(\lambda_{it}\right)=\gamma_{i}+x_{it}^{\prime }\beta ,\;i=1,...,N,\;t=1,...,T,\;$$ (4)

$$x_{it}^{\prime }\beta =\beta_{k}x_{kit},\;i=1,...,N,\;t=1,...,T,\;k=1,...,K,\;$$ (5)

where we assume there are K (k=1,…,K) independent variables for the *i*-th observation in the *t-*th period, and $\beta_{k}$ is the regression parameter for the *k-*th independent variable. It is noted that the attributes of each firm may influence the dependent variable, and the specific effects of each firm are described by $\gamma_{i}$, which is the time-invariant fixed effects term in Equation (6).


$$\gamma_{i}=\ln\left(\alpha_{i}\right),\;i=1,...,N.$$ (6)

Using the variables presented in Table 2 in Section 3.2.4, the estimated model in this study is formulated in Equation (7).


$$\ln\left({Innovation}_{it}\right)=\gamma_{i}$$

$$+f\left({FemaleYE}_{it},{\;Firm}_{it},{\;TMT}_{it},\;Yeardummy,Industrydummy\right)+e_{it},$$ (7)

where ${Innovation}_{it}$ is the number of innovations measured by *Patent Applications*, *Patents Granted*, *Citations*, and *Outside Citations*; ${FemaleYE}_{it}$ is the *Years of female employment*; ${Firm}_{it}$ is the firm characteristics;${\;TMT}_{it}$ is the TMT characteristics. All these variables are for the *i*-th firm and the t-th period. In addition, $e_{it}$ is a random error term and $\gamma_{i}$ is a fixed effects for the *i*-th observation. This study employs the fixed effects model that can avoid any distributional assumption of $e_{it}$ [106] so *t*hat we cannot include a time-invariant industry dummy as an independent variable to control industry-specific features. Thus, the industry dummy variable included in Equation (7) is only applied to the random effects model estimation for the robustness check against the fixed effects model in Section 4.2.

Negative Binomial (NB) regression is recommended for dealing with the overdispersion in case that the SDs are much larger than the means seen as our dependent variables. However, it is also known that the NB model with fixed effects for panel data is valid only under very restrictive distributional assumptions [107]. In contrast, Poisson regression with fixed effects is known to be valid under very general conditions, therefore we use the ppmlhdfe command on Stata 17 [108,109]. In addition, we conduct NB regression with fixed effects for the model in Section 4.2 for confirmation check as shown in Table A4.

## 4 Empirical Analysis and Results

### 4.1 Descriptive statistics and correlations

Table 3 presents the descriptive statistics of the sample. The average number of *Patent Applications* is 953, the average number of *Patents Granted* is 489, the average number of *Citations* is 891, and the average number of *Outside Citations* is 545. For example, in a previous study by Suzuki et al., the average number of patents filed by the top 10 companies in the Japanese electrical industry from 1991 to 2000 was 732 and the average number of claims was 4597 [110].

**Table 3:** Summary statistics.

	N	Mean	Max.	Min.	S.D.
Patent - Applications	2722	953.277	22487.000	0.000	1794.388
- Granted	2722	488.509	7979.000	0.000	855.971
Citations	2722	891.321	27198.000	0.000	2040.958
Outside Citations	2722	544.650	19807.000	0.000	1303.796
Years of Female Employment	2195	14.874	35.600	2.400	3.371
Firm Size	2687	9.974	12.860	6.047	1.131
ROA	2697	0.035	0.438	-0.519	0.046
Tangibility	2697	0.276	0.627	0.011	0.114
Leverage	2697	0.202	0.623	0.001	0.104
R&D Intensity	2693	0.034	0.229	0.000	0.030
Firm Age	2704	3.916	4.489	0.000	0.586
Female Ratio	2252	0.126	0.439	0.018	0.056
Years of Male Employment	2195	17.955	25.000	2.400	2.574
Directors Age	2662	4.096	4.390	3.833	0.047
CTO	2662	0.614	1.000	0.000	0.487
Female Directors ratio	2662	0.038	0.571	0.000	0.065
Outside Directors ratio	2662	0.225	1.000	0.000	0.164
Inventors	2722	2523.918	57614.000	0.000	4560.940
FIs	2722	5375.551	136198.000	0.000	11255.228
Themes	2722	2500.205	65459.000	0.000	4969.573
Claims	2722	6965.994	176606.000	0.000	14364.866

*Notes*: *N* exhibits firm years, 144 firms from 2004 to 2022. *N* is not even due to some missing data in the database. Max., Min., and S.D. represent the maximum, minimum, and standard deviations of the data.

Table 4 presents the correlation coefficients of the main variables. The numbers of *Patent Applications*, *Patents Granted*, *Citations*, and *Outside Citations* are all positively correlated with *Years of female employment*. Therefore, Hypothesis 1, “A positive relationship exists between years of female employment and innovation,” is supported. The other control variables are also significantly correlated with the dependent variables, indicating the need for multivariate analysis. The variance inflation factor (VIF) is calculated and is less than 1.68 for all VIF values. No multicollinearity problems are observed in the range [124].

**Table 4:** Pairwise Correlation.

	1	2	3	4	5	6	7	8	9	10	11	12	13	14	15	16	17	18	19	20	21
1. Patent Filed	1.000																				
																					
2. Patent Granted	0.949	1.000																			
	(0.000)																				
3. Citations	0.855	0.790	1.000																		
	(0.000)	(0.000)																			
4. Outside Citations	0.879	0.808	0.956	1.000																	
	(0.000)	(0.000)	(0.000)																		
5. Years of Female Employment	0.144	0.130	0.072	0.062	1.000																
	(0.000)	(0.000)	(0.001)	(0.003)																	
6. Firm Size	0.592	0.613	0.418	0.423	0.110	1.000															
	(0.000)	(0.000)	(0.000)	(0.000)	(0.000)																
7. ROA	-0.087	-0.081	-0.076	-0.073	-0.045	-0.109	1.000														
	(0.000)	(0.000)	(0.000)	(0.000)	(0.034)	(0.000)															
8. Tangibility	-0.109	-0.116	-0.031	-0.023	-0.057	-0.074	-0.178	1.000													
	(0.000)	(0.000)	(0.110)	(0.231)	(0.008)	(0.000)	(0.000)														
9. Leverage	0.046	0.057	0.039	0.039	0.081	0.233	-0.427	0.303	1.000												
	(0.018)	(0.003)	(0.044)	(0.041)	(0.000)	(0.000)	(0.000)	(0.000)													
10. R&D Intensity	0.220	0.207	0.170	0.177	0.043	0.170	0.035	-0.322	-0.194	1.000											
	(0.000)	(0.000)	(0.000)	(0.000)	(0.046)	(0.000)	(0.069)	(0.000)	(0.000)												
11. Firm Age	-0.005	0.009	-0.039	-0.038	0.056	0.059	-0.001	-0.093	0.032	0.130	1.000										
	(0.792)	(0.646)	(0.040)	(0.049)	(0.009)	(0.002)	(0.955)	(0.000)	(0.092)	(0.000)											
12. Female ratio	-0.060	-0.088	-0.075	-0.069	-0.027	-0.064	0.140	-0.246	-0.178	0.188	0.051	1.000									
	(0.005)	(0.000)	(0.000)	(0.001)	(0.212)	(0.002)	(0.000)	(0.000)	(0.000)	(0.000)	(0.016)										
13. Years of Male Employment	0.128	0.137	0.092	0.086	0.573	0.078	0.010	-0.013	0.070	-0.083	0.097	-0.098	1.000								
	(0.000)	(0.000)	(0.000)	(0.000)	(0.000)	(0.000)	(0.636)	(0.531)	(0.001)	(0.000)	(0.000)	(0.000)									
14. Directors Age	0.089	0.084	0.020	0.009	0.079	0.075	-0.024	0.075	-0.049	-0.139	0.063	-0.036	0.026	1.000							
	(0.000)	(0.000)	(0.299)	(0.630)	(0.000)	(0.000)	(0.219)	(0.000)	(0.012)	(0.000)	(0.001)	(0.091)	(0.226)								
15. CTO	0.153	0.161	0.155	0.134	-0.013	0.074	-0.061	0.122	0.032	-0.046	0.128	-0.176	-0.013	-0.017	1.000						
	(0.000)	(0.000)	(0.000)	(0.000)	(0.554)	(0.000)	(0.002)	(0.000)	(0.101)	(0.017)	(0.000)	(0.000)	(0.558)	(0.389)							
16. Female Directors ratio	-0.089	-0.117	-0.166	-0.161	0.013	0.152	0.153	-0.102	-0.002	0.004	0.045	0.299	-0.006	0.068	-0.136	1.000					
	(0.000)	(0.000)	(0.000)	(0.000)	(0.558)	(0.000)	(0.000)	(0.000)	(0.924)	(0.832)	(0.021)	(0.000)	(0.787)	(0.000)	(0.000)						
17. Outside Directors ratio	-0.134	-0.143	-0.239	-0.238	0.072	0.095	0.124	-0.186	0.019	0.163	0.130	0.285	-0.051	0.083	-0.188	0.573	1.000				
	(0.000)	(0.000)	(0.000)	(0.000)	(0.001)	(0.000)	(0.000)	(0.000)	(0.338)	(0.000)	(0.000)	(0.000)	(0.016)	(0.000)	(0.000)	(0.000)					
18. Inventors	0.974	0.938	0.820	0.837	0.152	0.614	-0.098	-0.106	0.072	0.194	0.020	-0.072	0.155	0.109	0.161	-0.067	-0.105	1.000			
	(0.000)	(0.000)	(0.000)	(0.000)	(0.000)	(0.000)	(0.000)	(0.000)	(0.000)	(0.000)	(0.307)	(0.001)	(0.000)	(0.000)	(0.000)	(0.000)	(0.000)				
19. FIs	0.986	0.926	0.885	0.906	0.136	0.543	-0.085	-0.102	0.043	0.230	-0.022	-0.044	0.101	0.067	0.146	-0.105	-0.146	0.942	1.000		
	(0.000)	(0.000)	(0.000)	(0.000)	(0.000)	(0.000)	(0.000)	(0.000)	(0.025)	(0.000)	(0.262)	(0.038)	(0.000)	(0.001)	(0.000)	(0.000)	(0.000)	(0.000)			
20. Themes	0.993	0.930	0.893	0.911	0.136	0.560	-0.086	-0.101	0.041	0.224	-0.013	-0.054	0.116	0.073	0.154	-0.103	-0.150	0.957	0.993	1.000	
	(0.000)	(0.000)	(0.000)	(0.000)	(0.000)	(0.000)	(0.000)	(0.000)	(0.032)	(0.000)	(0.486)	(0.010)	(0.000)	(0.000)	(0.000)	(0.000)	(0.000)	(0.000)	(0.000)		
21. Claim	0.962	0.895	0.832	0.861	0.160	0.547	-0.074	-0.118	0.022	0.250	-0.013	-0.027	0.092	0.104	0.144	-0.087	-0.111	0.913	0.966	0.966	1.000
	(0.000)	(0.000)	(0.000)	(0.000)	(0.000)	(0.000)	(0.000)	(0.000)	(0.251)	(0.000)	(0.507)	(0.197)	(0.000)	(0.000)	(0.000)	(0.000)	(0.000)	(0.000)	(0.000)	(0.000)	

*Notes:* This table reports the Pairwise correlations among major variables that are used in this study. *P*-values are in parentheses. Details on variables definitions and data sources are provided in Table 2.

### 4.2 Verification of Hypothesis 1: A positive relationship exists between years of female employment and innovation

Table 5 presents the estimation results of the panel data fixed effects Poisson regression with year and industry dummy variables. Column 1 presents *Patent Applications* as the dependent variable. The coefficient of *Years of female employment* is 0.035 and significant at the 1% level. In other words, firms with more years of female employment filed more patent applications. This coefficient 0.035 is multiplied by an exponential function to give an incidence rate ratio of 1.036, which implies the actual impact of the variable. Assuming that the other variables remain unchanged, a one-year increase in *Years of female employment* represents a 3.6% increase in *Patent Applications*. For every description of a case in which the explanatory variable is varied, it is assumed that the other variables remain unchanged. This finding supports Hypothesis 1, “A positive relationship exists between years of female employment and innovation.”

**Table 5:** The relation between female employees and innovation.

	(1) Patent Applications	(2) Patent Granted	(3) Citations	(4) Outside Citations	(5) Citations
Years of Female Employment	0.035***	0.027**	0.036***	0.042***	0.021**
	(0.012)	(0.011)	(0.013)	(0.015)	(0.010)
Firm Size	0.656***	0.551***	0.402***	0.391***	0.115
	(0.132)	(0.118)	(0.100)	(0.108)	(0.083)
ROA	0.335	0.740***	0.364	0.267	0.296
	(0.288)	(0.280)	(0.259)	(0.240)	(0.246)
Tangibility	-0.783	-0.560	-0.987	-0.585	-0.023
	(0.613)	(0.615)	(0.722)	(0.720)	(0.453)
Leverage	-0.691**	-0.652**	-0.651**	-0.783***	-0.170
	(0.274)	(0.315)	(0.278)	(0.232)	(0.251)
R&D Intensity	1.919	2.671	2.574	0.181	1.722
	(1.699)	(1.881)	(1.755)	(1.564)	(1.607)
Firm Age	-0.086**	-0.125**	-0.133***	-0.125***	-0.124***
	(0.040)	(0.061)	(0.033)	(0.041)	(0.029)
Female ratio	0.869	1.057	1.378*	2.018*	1.412**
	(0.863)	(0.881)	(0.830)	(1.106)	(0.679)
Years of Male Employment	-0.024*	-0.030**	-0.038**	-0.031*	-0.022
	(0.013)	(0.013)	(0.019)	(0.018)	(0.017)
Directors Age	1.015***	0.739*	0.807**	1.322**	0.673*
	(0.292)	(0.395)	(0.405)	(0.538)	(0.360)
CTO	0.067**	0.087**	-0.015	-0.001	-0.030
	(0.033)	(0.040)	(0.030)	(0.035)	(0.027)
Female Directors ratio	0.239	0.070	-0.151	-0.129	-0.090
	(0.316)	(0.310)	(0.434)	(0.465)	(0.386)
Outside Directors ratio	0.082	0.103	-0.161	-0.200	-0.264
	(0.159)	(0.184)	(0.227)	(0.251)	(0.223)
Patent Applications					0.000***
					(0.000)
Pseudo R2	0.969	0.964	0.974	0.973	0.977
N	2175	2175	2175	2175	2175

Notes: This table reports the results from panel data Poisson regressions of the number of patent applications, patents granted, citations and outside citations. Year and industry dummy variables are included. Superscripts ***, **, * represent significance at the 1%, 5%, and 10% levels, respectively. Robust standard errors clustered at the firm level are in parentheses. Details on variables definitions and data sources are provided in Table 2.

Column 2 presents the number of *Patents Granted* as the dependent variable. The coefficient of *Years of female employment* on *Patents granted* is 0.027 and significant at the 5% level. A one-year increase in *Years of female employment* represents a 2.7% (exp(0.027) =1.027) increase in the number of registered patents. The coefficient for *Years of male employment* is -0.030 and significant at the 5% level, which implies a one-year increase in *Years of male employment* (exp(-0.030) =0.970), representing a 3.0% decrease in the number of *Patents Granted*.

Column 3 shows *Citations* as the dependent variable. *Citations* represents a higher quality of innovation than does *Patents Granted*. The coefficient of *Years of female employment* is 0.036 and significant at the 1% level, which implies that a one-year increase in *Years of female employment* represents a 3.7% (exp(0.036) =1.037) increase in *Citations*. The increase in *Citations* is roughly the same as the increase in *Patents applications*. This implies that the increase in citations may be from a larger number of patent applications. We have tested with an offset for number of patents in the regression by adding *Patent Applications* as control variable as shown in Column 5. The coefficient of *Years of female employment* is still positive 0.021 and significant at the 5% level, which implies that a one-year increase in *Years of female employment* represents a 2.1% (exp(0.021) =1.021) increase in *Citations* with control of *Patent Applications*.

Column 4 presents *Outside Citations* as the dependent variable. *Outside Citations* represents a higher quality of innovation than does *Citations*. The coefficient of *Years of female employment* is 0.042 and significant at the 1% level, which implies that a one-year increase in *Years of female employment* represents a 4.3% (exp(0.042) =1.043) increase in *Outside Citations*.

The coefficients of *Female ratio* in Columns 1–2 are not significant, while those in Columns 3–4 are significant at the 10% level. The potential influence of the ratio of female employees on innovation is smaller than that of years of female employment.

In summary, a positive and significant relationship exists between *Years of female employment* and all four innovation indicators. These results support Hypothesis 1, which indicates that *years of female employment* is positively related to innovation.

To check the robustness of these results, we apply a random effects model instead of a fixed effects model, which includes an industry dummy variable as an independent variable, as described in Equation (7). The results are similar; the estimated coefficients for *Years of female employment* are (1) 0.035***, (2) 0.036***, (3) 0.042**, and (4) 0.035***, as shown in Table A2 in appendix. To address the concern about the serial correlation across the key independent variable of Years female employment, we perform simple Poisson regression after averaging all variables over time. The result shows a smaller but still positive coefficient for (mean) Years of female employment with (1) 0.035*, (2) 0.023**, (3) 0.041*, and (4) 0.038* (Table is not shown for simplicity). To address the concern that some of control variables may have non-linear relationships, we have tried to replace, for instance, two control variables of Years of male employment and Female Directors ratio with sets of dummy variables. After replacing Years of male employment with a dummy variable, the result shows a positive coefficient for Years of female employment with (1) 0.031***, (2) 0.028***, (3) 0.040***, and (4) 0.041***. After replacing Female Directors ratio with a dummy variable, the result shows a positive coefficient for Years of female employment with (1) 0.034***, (2) 0.024***, (3) 0.036***, and (4) 0.043***. (Tables are not shown for simplicity).

We also conduct the propensity score matching (PSM) by introducing a new dummy variable to exhibit the probability of firms having longer years of female employment, *D_HighFemaleYE*. If the gap between female and male years of employment is smaller than the total average gap (2.62 years), it is set to 1, otherwise 0. We match each treatment firm (*D_HighFemaleYE* = 1) with a control firm (*D_HighFemaleYE* = 0) to obtain 598 pairs, then run the same Poisson fixed effects model. The results show a smaller but still positive coefficient for *Years of female employment* with (1) 0.017, (2) 0.032**, (3) 0.032*, and (4) 0.035*, as shown in Table A3 in the appendix. These findings indicate that firms with longer female years of employment have a higher innovation performance compared to firms with shorter female years of employment. Thus, Hypothesis 1 is supported.

### 4.3 Verification of Hypothesis 2-1: A positive relationship exists between years of female employment and the breadth of technological field of innovation

Regarding one of the reasons why more years of female employment correlates positively with innovation, we hypothesize that female employees increase the breadth of technological field in the innovation process and promote higher-quality innovation. Previous studies have shown that firms with more technological holdings remain innovative and are long-term performers [82,83].

To test this hypothesis, we introduce three variables as indicators regarding the scope of technological research. First, we introduce the number of inventors (*Inventors*). A large number of inventors indicates that innovations are established by bringing together the technologies belonging to each inventor, and, thus, the scope of technological research is considered broad. Second, the number of FI technological fields identified for each patent is introduced (*FIs*). A large number of technology fields indicates that more technology fields are involved in the innovation process. Finally, we measure the number of claims (*Claims*). A higher number of claims indicates that more technological perspectives are considered in the innovation process.

Table 6, Column 1 shows *Inventors* as the dependent variable. The coefficient of *Years of female employment* is significant at the 1% level (0.031), which implies that a one-year increase in *Years of female employment* represents a 3.1% (exp(0.031) =1.031) increase in *Inventors*. Conversely, the coefficient for *Years of male employment* is -0.025 and significant at the 10% level, which implies a 2.5% (exp(0.025)=1.025) decrease in *Inventors* if employment of males is one year longer.

**Table 6:** The relation between female employees and technological diversity.

	(1) Inventors	(2) FIs	(3) Themes	(4) Claims
Years of Female Employment	0.031***	0.042***	0.045***	0.030*
	(0.012)	(0.013)	(0.012)	(0.016)
Firm Size	0.609***	0.764***	0.716***	0.832***
	(0.137)	(0.121)	(0.120)	(0.129)
ROA	0.190	0.330	0.380	0.608*
	(0.328)	(0.295)	(0.295)	(0.356)
Tangibility	-0.691	-0.661	-0.498	-0.716
	(0.577)	(0.576)	(0.630)	(0.780)
Leverage	-0.684**	-0.607**	-0.667***	-0.859***
	(0.328)	(0.255)	(0.256)	(0.296)
R&D Intensity	1.355	3.393*	2.548	3.779*
	(1.865)	(1.779)	(1.568)	(2.028)
Firm Age	-0.142***	-0.096**	-0.101***	-0.047
	(0.050)	(0.038)	(0.037)	(0.050)
Female ratio	0.817	0.417	0.862	-0.004
	(0.941)	(0.985)	(0.829)	(1.101)
Years of Male Employment	-0.025*	-0.036***	-0.032**	-0.012
	(0.013)	(0.013)	(0.013)	(0.015)
Directors Age	0.975***	0.929***	1.182***	1.954***
	(0.332)	(0.300)	(0.321)	(0.399)
CTO	0.060	0.031	0.065**	0.058
	(0.039)	(0.029)	(0.031)	(0.035)
Female Directors ratio	0.276	0.024	0.203	0.116
	(0.330)	(0.391)	(0.360)	(0.473)
Outside Directors ratio	0.041	0.117	0.160	0.083
	(0.192)	(0.183)	(0.171)	(0.223)
Pseudo R2	0.969	0.975	0.972	0.968
N	2175	2175	2175	2175

Notes: This table reports the results from panel data Poisson regressions of the number of inventors, FIs, theme codes, and claims. Year and industry dummy variables are included. Superscripts ***, **, * represent significance at the 1%, 5%, and 10% levels, respectively. Robust standard errors clustered at the firm level are in parentheses. Details on variables definitions and data sources are provided in Table 2.

Column 2 shows the *FI* technological fields as the dependent variable. The coefficient of *Years of female employment* is significant at the 1% level (0.042), which implies that a one-year increase in *Years of female employment* represents a 4.3% (exp(0.042) =1.043) increase in the *FI* technological fields. Conversely, the coefficient for *Years of male employment* is significant at the 1% level (-0.036), which implies a 3.5% (exp(-0.036)=0.965) decrease in the number of *FI* technological fields if employment of males is one year longer.

Column 3 shows the number of theme codes as the dependent variable. The coefficient of *Years of female employment* shows significance at the 1% level (0.045), implying that a one-year increase in *Years of female employment* represents an 4.6% (exp(0.045) =1.046) increase in *Themes*.

Column 4 shows *Claims* as the dependent variable. The coefficient of *Years of female employment* is significant at the 10% level (0.030), which implies that a one-year increase in *Years of female employment* represents an 3.0% (exp(0.030) =1.030) increase in *Claims*.

The coefficients of *Female ratio* in Columns 1–4 are all insignificant. The potential influence of the ratio of female employees to technological diversity is smaller than that of the years of female employment.

*Years of female employment* is positively and significantly related to inventors, FIs, and claims as indicators of the breadth of technological fields. Therefore, Hypothesis 2-1 is supported.

### 4.4 Validation of Hypothesis 2-2: Broad technology coverage mediates the relationship between years of female employment and high-impact innovation

We use mediation analysis to examine how female employees’ activities increase the scope of technological research and promote quality innovation. In general, the mediation effect satisfies the following conditions [111]. Condition (1): The explanatory variable (EV) significantly affects the dependent variable (DV) when there is no mediator. Condition (2): EV significantly affects the mediators. Condition (3): The mediator significantly affects DV. Condition (4): Incorporating the mediator into the analytical model reduces the impact of EV on DV.

*Years of female employment* is used as an EV to represent the activities of female employees. As mediators, *Inventors* is used in Columns 2 and 3, *FIs* in Columns 4 and 5, and *Claims* in Columns 6 and 7 of Table 7. *High-impact Patents* are used as DV and represent the number of innovations not based on previously held technologies or knowledge. Radical innovation requires the consideration of a wider range of technologies and having a spread of technologies is known to be more favorable for exploratory innovation [88]. Therefore, it can be used to indicate whether high-quality innovation has occurred.

**Table 7:** Mediation tests of Inventors, FIs, Themes and Claims as mediators on the relation between female employees and high-impact patents.

	(1) DV: High-impact Patent	(2) Mediator: Inventors	(3) DV: High-impact Patent	(4) Mediator: FIs	(5) DV: High-impact Patent	(6) Mediator: Themes	(7) DV: High-impact Patent	(8) Mediator: Claims	(9) DV: High-impact Patent
EV: Years of female employment	0.043***	0.031***	0.036***	0.042***	0.033***	0.045***	0.032***	0.030*	0.037***
	(0.016)	(0.012)	(0.011)	(0.013)	(0.013)	(0.012)	(0.012)	(0.016)	(0.011)
Mediator: Inventors			0.000***						
			(0.000)						
Mediator: FIs					0.000***				
					(0.000)				
Mediator: Themes							0.000***		
							(0.000)		
Mediator: Claim									0.000***
									(0.000)
Indirect Effect			16.5%		24.1%		26.2%		15.7%
Sobel Test (p-value)			0.017		0.003		0.001		0.079
All controls	YES	YES	YES	YES	YES	YES	YES	YES	YES
Pseudo R2	0.970	0.969	0.973	0.975	0.973	0.972	0.973	0.968	0.973
N	2175	2175	2175	2175	2175	2175	2175	2175	2175

Notes: This table reports the results from panel data Poisson regressions for mediation analyses. Column 1 shows High-impact patents (DV) and Years of female employment (EV). Columns 2, 4, 6 and 8 show the mediator (2; number of inventors, 4; number of FIs, 6; number of theme codes, 8; number of claims) and Years of female employment (EV). Columns 3, 5, 7, and 9 show DV and EV with mediators and the result of indirect effect and Sobel tests. Year and industry dummy variables are included. Superscripts ***, **, * represent significance at the 1%, 5%, and 10% levels, respectively. Robust standard errors clustered at the firm level are in parentheses. Details on variables definitions and data sources are provided in Table 2.

Table 7, Column 1 shows the relationship between DV and EV. The coefficient of *Years of female employment* is significant at the 1% level (0.043). Column 2 shows the relationship between EV and the mediator. The coefficient of *Years of female employment* is significant at the 1% level (0.031), which implies that a one-year increase in the number of years of the employment of females represents a 3.1% increase in *Inventors*, which is the same result shown in Table 6, column 1.

Column 3 shows the results with the addition of the mediator. The coefficient of *Inventors* is significant at the 1% level at 0.000 (0.0000296). For example, an increase of the total number of 1000 inventors represents a 3.0% increase (exp(0.0000296*1000) =1.030) in the number of high-impact patents. The coefficient of *Years of female employment* is significant at the 1% level (0.036). This is a decrease of 0.007 relative to the coefficient of 0.043 without the mediator in Column 1. This satisfies condition (4). A Sobel test is performed for the mediator and the p-value is statistically significant at the 5% level. Further, as shown in Column 3, the mediating effect is (0.043–0.036)/0.043 = 16.5% (Sobel test, p = 0.017).

Column 4 shows the results of the number of FIs, and the coefficient of *Years of female employment* is significant at the 1% level (0.042). As shown in Column 5, the coefficient of the number of FIs is significant at the 1% level at 0.000 (0.00001003). For example, an increase of 1000 FIs represents a 1.0% increase (exp(0.00001003*1000 = 1.010) in the number of high-impact patents. The mediation effect is 24.1% (Sobel test, p = 0.003).

Columns 6 and 7 show the results for the number of theme codes, which represent the number of technical fields. As shown in Column 7, the coefficient of *Themes* is significant at the 1% level at 0.000 (0.0000217). For example, an increase of 1000 theme codes represents a 2.2% increase (exp(0.0000217*1000 = 1.022) in the number of high-impact patents. The mediation effect is 26.1% (Sobel test, p = 0.001).

Columns 8 and 9 show the results for the number of claims. As shown in Column 9, the coefficient of *Claims* is significant at the 1% level at 0.000 (0.00000752). For example, an increase of 1000 claims represents a 0.7% increase (exp(0.00000752*1000 = 1.007) in the number of high-impact patents. The mediation effect is 15.7% (Sobel test, p = 0.079).

Among the mediators, the number of theme codes has the highest mediation effect (26.2%). This implies that the number of female employees is most strongly associated with the number of theme codes, and theme codes is most strongly associated with high-impact innovation.

Taken together, these findings suggest that there is also a pathway through which firms employing female employees for longer periods have a wider technological field and, consequently, a greater number of high-impact patents. Hypothesis 2-2 is therefore supported.

## 5 Discussion and Conclusion

This study investigated how female employees contribute to firm technological innovation by applying panel data Poisson regression models to data pertaining to 144 Japanese listed manufacturing firms from 2004 to 2022. The research yielded three findings.

First, longer female employment was found to be positively related to innovation. This encourages Japanese management bodies to aware that women’s contributions can be helpful in their organizations’ innovation. It also indicates that employees in positions below the leadership level could have a significant impact on innovation. This might be linked to the family or “bottom-up approach” cultures in Japanese firms. A survey reported that longer female employment could be attributed to female employees feeling more secure at their workplaces [112]. Further, longer male employment was found to be negatively related to innovation. This is possibly due to the workplace being male-dominant or because of the Japanese culture of excluding outsiders. Our results imply that innovation can be promoted by increasing employee diversity and their interactions with each other.

Second, longer female employment was found to be positively related to the number of technological fields. This could be because females participate in innovation processes with different technical backgrounds or research styles from those of males [113–116]. In addition, women’s diverse perspectives [9,85,86], communication skills [59,117], and team-oriented work styles [87] could influence the broadening of technological fields. Mauleón and Bordons have found that women tend to collaborate more than men, according to their analysis of Spanish inventors from 1990–2005 [87]. Our finding concerning longer female employment might be due to this same reason of a higher female share in highly collaborative environments.

Third, this study presented the mediating effects of technological fields on the relationship between high-impact innovation and the years of female employment. The evidence that longer female employment can help widen the technological field and promote high-impact innovation might be consistent with the unique female characteristics mentioned above. Interestingly, this relationship is important for companies because firms oriented toward exploratory innovation are likelier to take risks for future growth [57]. Japanese firms are good at exploitation. One of the reasons why Japanese firms entered a long and dark tunnel following the Japanese economic bubble crash in 1989 was that they had not recognized the importance of ambidexterity, the causation for the exploitation of existing business versus effectuation for exploring new business [118].

Based on these findings, we encourage Japanese corporate management to create a system that allows female employees to play an active role in the workplace by enhancing their long-term employment, encouraging gender diverse interactions among employees, and promoting innovation within the firm. Female employees’ diverse perspectives, communication skills, and team orientation would be helpful to facilitate a broader range of technological research. Utilizing these characteristics of female employees could make it easy to realize high-impact innovation or high-quality innovation, both of which are particularly difficult to achieve because of their higher probability of failure. It could also help balance difficult, new exploration skills with existing exploitation skills, which can be a key strategy for future growth [119].

This study has several limitations. First, the number of inventors and the number of claims may not directly correspond to technology breadth, because the setting criteria of these variables might be affected based on each R&D team or management unit. Further study will be needed for the precise estimation. Second, this study used data on Japan’s major manufacturing firm from the Nikkei Stock Average, a representative stock indicator. It remains to be verified whether the same trend holds for smaller firms, non-listed firms, or industries other than manufacturing. Third, although patent information was used to measure degrees of innovation, not all innovations are patented, and innovations involving trade secrets or know-how are not covered in this study. Fourth, we prepared the panel data and conducted robustness tests to address the concern about continued unobserved heterogeneity. However, there remains the possibility of omitted variable bias regarding both years of female employment and innovation, including bias from the omission of a variable such as firm culture. Fifth, this study did not examine whether female employees directly influence innovation as members of an R&D team or indirectly contribute to innovation such as through an IP department. Developing a more detailed classification of the attributes of female employees is a topic for future research. Sixth, we found that the number of years of male employment have a negative relation with innovation. The mechanism behind this opposite effect of males and females requires further exploration. These are all future tasks of this study.

## Supporting information

S1 Text Appendix A. (DOCX)

S1 Data Anonymized data. (XLSX)
