# Supplementary material for: Relationship between female employees and firm’s innovation: Evidence from Japanese companies
Source: PLoS One. 2025 Jun 3;20(6):e0323751. doi: 10.1371/journal.pone.0323751 (PMC12133021; doi:10.1371/journal.pone.0323751)
Supplement: S1 Text — (DOCX) [file pone.0323751.s001.docx]

**Appendix A:** Ratio of female employees, years of female employment

The ratio of female employees and years of female employment data are obtained from the CSR database. They are summarized in Table A by year, industry, and age group. The proportion of women increased from 11% in 2004 (lowest) to 15% in 2022 (highest). On the other hand, years of female employment was 14.02 years in 2004 and peaked at 15.44 years in 2018. The average years of female employment (14.82) is nearly 3 years less than that of male (17.98) for the 2004–2022 period.

The highest ratio of females (0.20) is found in the pharmaceutical industry (Industry Code 16a), and the lowest ratio of females (0.06) is found in the shipbuilding industry (code 30). The highest years of female employment (21.24 years) are found in the toy industry (Code 32 as others), and the lowest years of female employment (9.56 years) are found in the printing industry (code 15). With an average age of 25 years, the highest female ratio is 0.17 and years of female employment is 11.5 years. With an average age of 30 years, the female ratio is 0.13 and years of female employment is 7.86 years at the lowest. Considering the results for both age groups, some firms may expect female employees to leave the company in their mid-30s, and may intend to benefit from providing lower wages to younger female employees.

With an average age of 45 years, the years of female employment is the highest (21.69) among firms. The largest number of observations (290) had an average female age of 40, a *female ratio* of 0.13, and years of female employment of 16.20 years. The number of years of employment increased with the average employee age.

| **Table A1: Distribution of female employees in comparison with male employees** | | | | | | | | | | | | | | | | | | | | | | |
| --- | --- | --- | --- | --- | --- | --- | --- | --- | --- | --- | --- | --- | --- | --- | --- | --- | --- | --- | --- | --- | --- | --- |
| **Panel A: By year** |  |  |  |  |  |  |  |  |  |  |  |  |  |  |  |  |  |  |  |  |  |  |
| Year | 2004 | 2005 | 2006 | 2007 | 2008 | 2009 | 2010 | 2011 | 2012 | 2013 | 2014 | 2015 | 2016 | 2017 | 2018 | 2019 | 2020 | 2021 | 2022 |  | Total | Ave. |
| N | 141 | 141 | 141 | 142 | 143 | 143 | 143 | 144 | 144 | 144 | 144 | 144 | 144 | 144 | 144 | 144 | 144 | 144 | 144 |  | 2722 |  |
| Female Ratio | 0.11 | 0.12 | 0.11 | 0.11 | 0.11 | 0.11 | 0.12 | 0.12 | 0.12 | 0.12 | 0.12 | 0.12 | 0.13 | 0.13 | 0.13 | 0.14 | 0.14 | 0.14 | 0.15 |  |  | 0.12 |
| Distribution | 0.05 | 0.05 | 0.05 | 0.05 | 0.05 | 0.05 | 0.05 | 0.05 | 0.05 | 0.05 | 0.05 | 0.05 | 0.05 | 0.05 | 0.05 | 0.05 | 0.05 | 0.05 | 0.05 |  | 1.00 |  |
| N | 77 | 89 | 96 | 99 | 102 | 104 | 109 | 113 | 119 | 121 | 125 | 126 | 130 | 129 | 130 | 133 | 132 | 131 | 130 |  | 2195 |  |
| Years of female employment | 14.02 | 14.24 | 14.73 | 14.73 | 14.55 | 14.34 | 14.17 | 14.43 | 14.58 | 14.61 | 15.13 | 15.31 | 15.29 | 15.37 | 15.44 | 15.22 | 15.22 | 15.03 | 15.19 |  |  | 14.82 |
| Distribution | 0.04 | 0.04 | 0.04 | 0.05 | 0.05 | 0.05 | 0.05 | 0.05 | 0.05 | 0.06 | 0.06 | 0.06 | 0.06 | 0.06 | 0.06 | 0.06 | 0.06 | 0.06 | 0.06 |  | 1.00 |  |
| N | 77 | 89 | 96 | 99 | 102 | 104 | 109 | 113 | 119 | 121 | 125 | 126 | 130 | 129 | 130 | 133 | 132 | 131 | 130 |  | 2195 |  |
| Years of male employment | 18.46 | 18.54 | 18.61 | 18.42 | 18.12 | 17.73 | 17.58 | 17.42 | 17.54 | 17.55 | 17.84 | 17.83 | 17.88 | 18.02 | 17.96 | 17.95 | 17.93 | 18.02 | 18.21 |  |  | 17.98 |
| Distribution | 0.04 | 0.04 | 0.04 | 0.05 | 0.05 | 0.05 | 0.05 | 0.05 | 0.05 | 0.06 | 0.06 | 0.06 | 0.06 | 0.06 | 0.06 | 0.06 | 0.06 | 0.06 | 0.06 |  | 1.00 |  |
| **Panel B: By industry** |  |  |  |  |  |  |  |  |  |  |  |  |  |  |  |  |  |  |  |  |  |  |
| Industry Code | 04 | 05 | 06 | 09 | 11 | 14 | 15 | 16 | 16a | 17 | 19 | 21 | 22 | 23 | 27 | 28 | 29 | 30 | 31 | 32 | Total | Ave. |
| N | 19 | 19 | 171 | 206 | 38 | 31 | 57 | 323 | 171 | 34 | 38 | 152 | 76 | 152 | 304 | 114 | 570 | 38 | 190 | 19 | 2722 |  |
| Female Ratio | 0.15 | 0.14 | 0.12 | 0.19 | 0.13 | 0.11 | 0.16 | 0.12 | 0.20 | 0.11 | 0.06 | 0.12 | 0.06 | 0.10 | 0.09 | 0.14 | 0.13 | 0.06 | 0.08 | 0.16 |  | 0.12 |
| Distribution | 0.01 | 0.01 | 0.06 | 0.08 | 0.01 | 0.01 | 0.02 | 0.12 | 0.06 | 0.01 | 0.01 | 0.06 | 0.03 | 0.06 | 0.11 | 0.04 | 0.21 | 0.01 | 0.07 | 0.01 | 1.00 |  |
| N | 15 | 15 | 151 | 175 | 38 | 21 | 55 | 290 | 124 | 22 | 37 | 95 | 47 | 90 | 234 | 96 | 490 | 19 | 163 | 18 | 2195 |  |
| Years of female employment | 16.02 | 11.51 | 13.67 | 14.56 | 14.10 | 17.07 | 9.56 | 15.45 | 14.75 | 17.82 | 12.98 | 15.99 | 17.33 | 14.48 | 13.69 | 14.41 | 16.05 | 15.17 | 13.87 | 21.24 |  | 14.99 |
| Distribution | 0.01 | 0.01 | 0.07 | 0.08 | 0.02 | 0.01 | 0.03 | 0.13 | 0.06 | 0.01 | 0.02 | 0.04 | 0.02 | 0.04 | 0.11 | 0.04 | 0.22 | 0.01 | 0.07 | 0.01 | 1.00 |  |
| N | 15 | 15 | 151 | 175 | 38 | 21 | 55 | 290 | 124 | 22 | 37 | 95 | 47 | 90 | 234 | 96 | 490 | 19 | 163 | 18.00 | 2195 |  |
| Years of male employment | 16.67 | 16.11 | 17.50 | 18.21 | 15.96 | 19.92 | 14.80 | 18.12 | 17.81 | 20.26 | 16.45 | 19.01 | 18.99 | 18.42 | 17.41 | 18.48 | 18.03 | 16.74 | 18.37 | 21.26 |  | 17.93 |
| Distribution | 0.01 | 0.01 | 0.07 | 0.08 | 0.02 | 0.01 | 0.03 | 0.13 | 0.06 | 0.01 | 0.02 | 0.04 | 0.02 | 0.04 | 0.11 | 0.04 | 0.22 | 0.01 | 0.07 | 0.01 | 1.00 |  |
| **Panel C: By Age** |  |  |  |  |  |  |  |  |  |  |  |  |  |  |  |  |  |  |  |  |  |  |
| Average Age* | 25 | 29 | 30 | 31 | 32 | 33 | 34 | 35 | 36 | 37 | 38 | 39 | 40 | 41 | 42 | 43 | 44 | 45 | 46 |  | Total | Ave. |
| N | 1 | 6 | 15 | 16 | 43 | 54 | 87 | 141 | 202 | 246 | 267 | 275 | 290 | 237 | 128 | 115 | 54 | 9 | 1 |  | 2187 |  |
| Female Ratio | 0.17 | 0.12 | 0.13 | 0.13 | 0.12 | 0.12 | 0.11 | 0.12 | 0.11 | 0.12 | 0.13 | 0.13 | 0.13 | 0.15 | 0.14 | 0.13 | 0.13 | 0.10 | 0.15 |  |  | 0.13 |
| Distribution | 0.00 | 0.00 | 0.01 | 0.01 | 0.02 | 0.02 | 0.04 | 0.06 | 0.09 | 0.11 | 0.12 | 0.13 | 0.13 | 0.11 | 0.06 | 0.05 | 0.02 | 0.00 | 0.00 |  | 1.00 |  |
| N | 1 | 6 | 15 | 16 | 43 | 54 | 87 | 139 | 201 | 243 | 267 | 274 | 290 | 235 | 128 | 115 | 54 | 9 | 1 |  | 2178 |  |
| Years of female employment | 11.50 | 8.42 | 7.86 | 9.03 | 9.55 | 10.52 | 11.60 | 12.29 | 13.07 | 13.52 | 14.13 | 15.25 | 16.20 | 16.88 | 17.96 | 19.57 | 20.49 | 21.69 | 21.00 |  |  | 14.24 |
| Distribution | 0.00 | 0.00 | 0.01 | 0.01 | 0.02 | 0.02 | 0.04 | 0.06 | 0.09 | 0.11 | 0.12 | 0.13 | 0.13 | 0.11 | 0.06 | 0.05 | 0.02 | 0.00 | 0.00 |  | 1.00 |  |
| N | 1 | 6 | 15 | 16 | 43 | 54 | 87 | 139 | 201 | 243 | 267 | 274 | 290 | 235 | 128 | 115 | 54 | 9 | 1 |  | 2178 |  |
| Years of male employment | 17.10 | 16.13 | 15.06 | 15.10 | 15.77 | 16.45 | 17.42 | 17.33 | 17.61 | 17.75 | 17.92 | 17.88 | 18.05 | 18.57 | 18.57 | 19.68 | 20.01 | 20.74 | 20.20 |  |  | 17.76 |
| Distribution | 0.00 | 0.00 | 0.01 | 0.01 | 0.02 | 0.02 | 0.04 | 0.06 | 0.09 | 0.11 | 0.12 | 0.13 | 0.13 | 0.11 | 0.06 | 0.05 | 0.02 | 0.00 | 0.00 |  | 1.00 |  |
| Note: Average female age has some missing data; therefore, the number of total observations is not the same as in Panels A and B. | | | | | | | | | | | | | | | | | | | | | | |

**Figure A1**: The trend of the proportion of female employees

**Figure A2**: Changes in the average years of female employment

| **Table A2: The relation between female employees and innovation (Random effects)** | | | | |
| --- | --- | --- | --- | --- |
|  | (1) Patent Applications | (2) Patent Granted | (3) Citations | (4) Outside Citations |
| Years of Female Employment | 0.035*** | 0.036*** | 0.042*** | 0.035*** |
|  | (0.012) | (0.013) | (0.015) | (0.012) |
| Firm Size | 0.657*** | 0.405*** | 0.397*** | 0.657*** |
|  | (0.131) | (0.099) | (0.106) | (0.131) |
| ROA | 0.336 | 0.365 | 0.269 | 0.336 |
|  | (0.286) | (0.258) | (0.240) | (0.286) |
| Tangibility | -0.782 | -0.989 | -0.587 | -0.782 |
|  | (0.609) | (0.718) | (0.715) | (0.609) |
| Leverage | -0.691** | -0.651** | -0.783*** | -0.691** |
|  | (0.273) | (0.277) | (0.231) | (0.273) |
| R&D Intensity | 1.917 | 2.577 | 0.186 | 1.917 |
|  | (1.690) | (1.749) | (1.556) | (1.690) |
| Firm Age | -0.086** | -0.133*** | -0.124*** | -0.086** |
|  | (0.039) | (0.033) | (0.041) | (0.039) |
| Female ratio | 0.867 | 1.377* | 2.016* | 0.867 |
|  | (0.860) | (0.825) | (1.098) | (0.860) |
| Years of Male Employment | -0.024* | -0.038** | -0.031* | -0.024* |
|  | (0.013) | (0.019) | (0.018) | (0.013) |
| Directors Age | 1.015*** | 0.806** | 1.319** | 1.015*** |
|  | (0.291) | (0.404) | (0.537) | (0.291) |
| CTO | 0.067** | -0.015 | -0.002 | 0.067** |
|  | (0.033) | (0.030) | (0.034) | (0.033) |
| Female Directors ratio | 0.239 | -0.149 | -0.125 | 0.239 |
|  | (0.315) | (0.432) | (0.462) | (0.315) |
| Outside Directors ratio | 0.082 | -0.161 | -0.201 | 0.082 |
|  | (0.158) | (0.226) | (0.250) | (0.158) |
| Log pseudolikelihood | -64980.859 | -63491.162 | -43112.151 | -64980.859 |
| chi2 | 50626.006 | 1149196.204 | 269672.766 | 50626.006 |
| N | 2177 | 2177 | 2177 | 2177 |
| Notes: This table reports the results from panel data Poisson regressions of the number of patent applications, patents granted, citations, and outside citations. Year and industry dummy variables are included. Superscripts ***, **, * represent significance at the 1%, 5%, and 10% levels, respectively. Robust standard errors clustered at the firm level are in parentheses. Details on variables definitions and data sources are provided in Table 2. | | | | |

| **Table A3: The relation between female employees and innovation** | | | | | |
| --- | --- | --- | --- | --- | --- |
|  | (0) D_HighFemaleYE | (1) Patent Applications | (2) Patent Granted | (3) Citations | (4) Outside Citations |
| Years of Female Employment |  | 0.017 | 0.032** | 0.032* | 0.035* |
|  |  | (0.012) | (0.013) | (0.017) | (0.020) |
| Firm Size | 0.108 | 0.656*** | 0.588*** | 0.473*** | 0.401*** |
|  | (0.725) | (0.140) | (0.130) | (0.115) | (0.124) |
| ROA | -0.623 | 0.514* | 0.709** | 0.474* | 0.408* |
|  | (2.528) | (0.286) | (0.294) | (0.278) | (0.227) |
| Tangibility | -3.891 | -1.051* | -1.126* | -1.618** | -0.804 |
|  | (2.611) | (0.612) | (0.589) | (0.806) | (0.794) |
| Leverage | 1.201 | -1.179*** | -1.335*** | -0.906** | -1.055*** |
|  | (2.100) | (0.229) | (0.275) | (0.385) | (0.336) |
| R&D Intensity | -24.271** | 3.351** | 5.890*** | 5.840** | 2.284 |
|  | (11.953) | (1.609) | (1.923) | (2.349) | (1.862) |
| Firm Age | 0.807 | 0.060 | -0.037 | -0.032 | -0.007 |
|  | (0.605) | (0.119) | (0.147) | (0.065) | (0.074) |
| Female ratio | -32.864*** | -2.493* | 0.090 | 1.200 | 1.226 |
|  | (6.528) | (1.479) | (1.626) | (1.738) | (1.945) |
| Years of Male Employment | -0.558*** | -0.036* | -0.044*** | -0.050** | -0.051** |
|  | (0.073) | (0.020) | (0.017) | (0.024) | (0.023) |
| Directors Age | 8.510** | 0.853 | 0.546 | 0.658 | 0.987 |
|  | (3.893) | (0.610) | (0.766) | (0.629) | (0.664) |
| CTO | 0.165 | 0.033 | 0.056 | -0.038 | -0.035 |
|  | (0.224) | (0.040) | (0.041) | (0.039) | (0.056) |
| Female Directors ratio | -0.340 | 0.120 | -0.210 | -0.198 | -0.129 |
|  | (2.135) | (0.334) | (0.276) | (0.412) | (0.506) |
| Outside Directors ratio | -1.668 | -0.016 | 0.138 | -0.534* | -0.567* |
|  | (1.173) | (0.161) | (0.190) | (0.308) | (0.311) |
| Pseudo R2 | 0.432 | 0.977 | 0.971 | 0.974 | 0.973 |
| N | 1385 | 1196 | 1196 | 1196 | 1196 |
| Notes: This table reports the results from the logistic regression of *D_HighFemaleYE* (Column 0), then each treatment firm (*D_HighFemaleYE*=1) with a control firm (*D_HighFemaleYE*=0) is matched based on a similar propensity score using caliper 0.01 with no replacement. Columns 1–4 show the results of Poisson regression of matched 598 pairs for the number of patent applications, patents granted, citations and outside citations. Year and industry dummy variables are included. Superscripts ***, **, * represent significance at the 1%, 5%, and 10% levels, respectively. Robust standard errors clustered at the firm level are in parentheses. Details on variables definitions and data sources are provided in Table 2. | | | | | |

| **Table A4** | | | | |
| --- | --- | --- | --- | --- |
| **The relation between female employees and innovation (negative binomial regression)** | | | | |
|  | (1) Patent Applications | (2) Patent Granted | (3) Citations | (4) Outside Citations |
| Years of Female Employment | 0.017*** | 0.018*** | 0.014** | 0.018*** |
|  | (0.005) | (0.005) | (0.006) | (0.006) |
| Firm Size | 0.326*** | 0.288*** | 0.407*** | 0.446*** |
|  | (0.031) | (0.031) | (0.030) | (0.030) |
| ROA | 0.344 | 0.592*** | 0.541** | 0.389* |
|  | (0.215) | (0.214) | (0.215) | (0.228) |
| Tangibility | -0.052 | 0.050 | 0.057 | 0.099 |
|  | (0.169) | (0.171) | (0.191) | (0.203) |
| Leverage | -0.436*** | -0.422*** | -0.392*** | -0.349** |
|  | (0.126) | (0.125) | (0.139) | (0.146) |
| R&D Intensity | 0.796 | 0.856 | -1.094 | -0.994 |
|  | (0.729) | (0.730) | (0.772) | (0.830) |
| Firm Age | -0.074** | -0.029 | -0.108*** | -0.085** |
|  | (0.037) | (0.045) | (0.036) | (0.038) |
| Female ratio | 0.735** | 1.464*** | 0.343 | 0.018 |
|  | (0.350) | (0.353) | (0.382) | (0.418) |
| Years of Male Employment | -0.017*** | -0.024*** | -0.012** | -0.012* |
|  | (0.005) | (0.005) | (0.006) | (0.006) |
| Directors Age | 1.509*** | 1.310*** | 1.089*** | 1.285*** |
|  | (0.220) | (0.223) | (0.265) | (0.287) |
| CTO | 0.026 | 0.053*** | -0.023 | -0.012 |
|  | (0.019) | (0.019) | (0.021) | (0.023) |
| Female Directors ratio | 0.302* | 0.042 | -0.022 | -0.073 |
|  | (0.167) | (0.173) | (0.209) | (0.228) |
| Outside Directors ratio | -0.314*** | -0.402*** | -0.359*** | -0.524*** |
|  | (0.093) | (0.094) | (0.103) | (0.112) |
| Log-likelihood | -12708.728 | -11242.064 | -11804.105 | -10429.780 |
| N | 2175 | 2175 | 2175 | 2175 |
| Notes: This table reports the results from panel data negative binomial regressions of the number of patent applications, patents granted, citations and outside citations. Year and industry dummy variables are included. Superscripts ***, **, * represent significance at the 1%, 5%, and 10% levels, respectively. Standard errors are in parentheses. Details on variables definitions and data sources are provided in Table 2. | | | | |
